# Bone mineral density changes around the stem correlate with stress changes after total hip arthroplasty: A study using thermoelastic stress analysis

**DOI:** 10.1002/jeo2.12031

**Published:** 2024-05-13

**Authors:** Ryunosuke Watanabe, Hajime Mishima, Hironori Takehashi, Hiroshi Wada, Sho Totsuka, Tomofumi Nishino, Masashi Yamazaki

**Affiliations:** ^1^ Department of Orthopaedic Surgery, Institute of Medicine University of Tsukuba Tsukuba Japan

**Keywords:** bone mineral density, stress distribution, thermoelastic stress analysis, total hip arthroplasty

## Abstract

**Purpose:**

Thermoelastic stress analysis (TSA) was used to evaluate stress changes over the entire surface of a specimen. This study aimed to assess the relationship between femoral stress distribution, analysed using TSA and changes in bone mineral density (BMD) after total hip arthroplasty (THA).

**Methods:**

Stress changes in the simulated bone before and after taper‐wedge stem insertion were measured using the TSA. Stress changes were compared with BMD changes around the stem 1 year after surgery in a THA patient (58 hips) with the same taper‐wedge stem. Subsequently, we compared the correlation between stress changes and BMD changes.

**Results:**

TSA revealed significant stress changes before and after stem insertion, with prominent alterations in the proximal medial region. The BMD changes at 1 year post‐THA exhibited a 15%–25% decrease in the proximal zones, while Zones 2–6 showed a −6% to 3% change. Notably, a strong positive correlation (0.886) was found between the stress change rate and BMD change rate.

**Conclusions:**

This study demonstrated a high correlation between femoral stress distribution assessed using TSA and subsequent BMD changes after THA. The TSA method offers the potential to predict stress distribution and BMD alterations postsurgery, aiding in implant development and clinical assessment. Combining TSA with finite element analysis could provide even more detailed insights into stress distribution.

**Level of Evidence:**

Case series (with or without comparison).

**Abbreviations**
BMD: bone mineral density
BMI: body mass index
DEXA: dual‐energy X‐ray absorptiometry
FEA: finite element analysis
HA: hydroxyapatite
THA: total hip arthroplasty
TSA: thermoelastic stress analysis

## INTRODUCTION

Total hip arthroplasty (THA) is performed worldwide, with good long‐term results owing to recent improvements in implants and surgical techniques. However, challenges such as aseptic loosening, infection, bone atrophy, dislocation and periprosthetic fractures remain after THA, which affect long‐term outcomes [26]. Stress transfer in the femur after THA depends on the stem size, design, material and whether the stem is cemented or cementless. Femoral remodelling occurs in response to variations in stress transfer [12, 21]. Bone atrophy due to stress shielding, particularly in the proximal femur, remains an unresolved problem [5]. Therefore, to achieve stress transfer to the proximal femur and bone preservation, implant design has been studied and femoral stress analysis has been performed.

Conventional femoral stress analysis studies use strain gauge‐based methods [1, 17, 19, 25]. However, this method can only measure the strain at the point where the gauge is used; therefore, it is arduous to observe the entire femur. Reports indicate that finite element analysis (FEA) using computed tomography (CT) data is a useful method for evaluating stress distribution and stem fixation of the femur after THA [10, 11, 23, 27, 32]. In recent years, as computer performance has improved, FEA has become widely used. However, because FEA is a numerical model based on simulations, it is important to verify and evaluate it using experimental models as well [19, 28]. Therefore, Hyodo et al. reported a method that enables noncontact observation of the stress distribution over the entire simulated bone surface under load using the thermoelastic stress imaging method [13]. This method is the only technique that helps in the observation of the stress distribution over the entire simulated bone in a mechanical experiment. However, to the best of our knowledge, there have been no reports comparing the stress distribution obtained using this technique with the clinical results after THA.

Hence, this novel study examined whether the stress distribution obtained using thermoelastic stress analysis (TSA) is related to the stress distribution after THA by correlating the stress change in the simulated bone with the change in the bone mineral density (BMD) around the stem.

## MATERIALS AND METHODS

### Thermoelastic stress analysis

#### Materials

**Figure 1:**
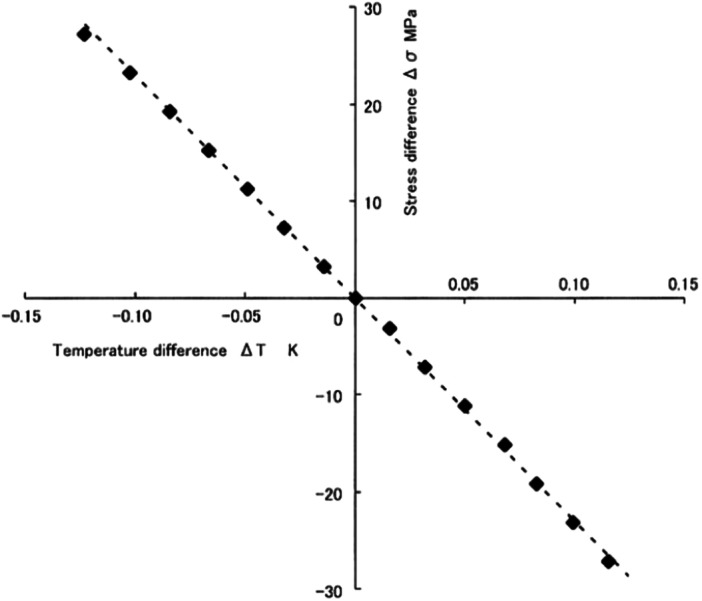
Thermoelastic property of the simulated bone (Hyodo et al., [13]).

A simulated femur was used in this experiment (Composite femur® #3403, Pacific Research Laboratories). The simulated femur is made of glass‐filled epoxy and mimics the mechanical properties and morphology of the adult male biological femur. Furthermore, it is composed of cortical and trabecular bone in structure [8, 9]. The thermoelastic properties of the glass‐filled epoxy used for cortical bone have a linear relationship between the change in the sum of principal stresses and temperature change. A temperature change of 1 K corresponds to approximately 227 MPa of change in the sum of principal stresses [13] (Figure 1). The kelvin (K) is the unit of thermodynamic temperature. A temperature of 0 K represents absolute zero, the absence of all heat. A change of 1°C is equivalent to that of 1 K.

We used an Anthology (Smith & Nephew), a taper‐wedge stem coated with hydroxyapatite (HA) in the proximal portion. Additionally, a 32 mm diameter OXINIUM femoral head (Smith & Nephew) was used.

#### Thermoelastic stress imaging method

The thermoelastic effect is the temperature change associated with a change in the stress of an object in an adiabatic state and is expressed by Equation (1), which was proposed by Thomson in 1853 [30].

(1) $$\Delta T=-kT\Delta (\sigma_{1}+\sigma_{2}).$$

Δ*T* is the temperature change (K), *k* is the thermoelastic constant of the material (1/Pa), *T* is the absolute temperature of the material (*K*) and *Δ*(*σ*_1_ + *σ*_2_) is the sum of principal stresses (Pa).

The thermoelastic constant, *k*, is given by

k=α/(ρCp),

*α* is the Coefficient of linear thermal expansion (K^−1^), *ρ* is the density (kg/m^3^) and *Cp* is the coefficient of specific heat at constant pressure of the material (J/(kg･K)).

The thermoelastic stress imaging method is based on the measurement of a slight temperature change (*ΔT*) that occurs when a material is subjected to elastic cyclic loading. Subsequently, the temperature change was converted into a change in the sum of the principal stresses using Equation (1). However, it was not possible to measure the principal stress components *σ*1 and *σ*2 separately. A high‐precision infrared thermography camera was used for measurements that have been applied not only in the industrial field but also in the evaluation of orthopaedic implants and the measurement of stress distribution in the femur [24, 33].

#### Setting of simulated bone and thermoelastic stress image measurement

**Figure 2:**
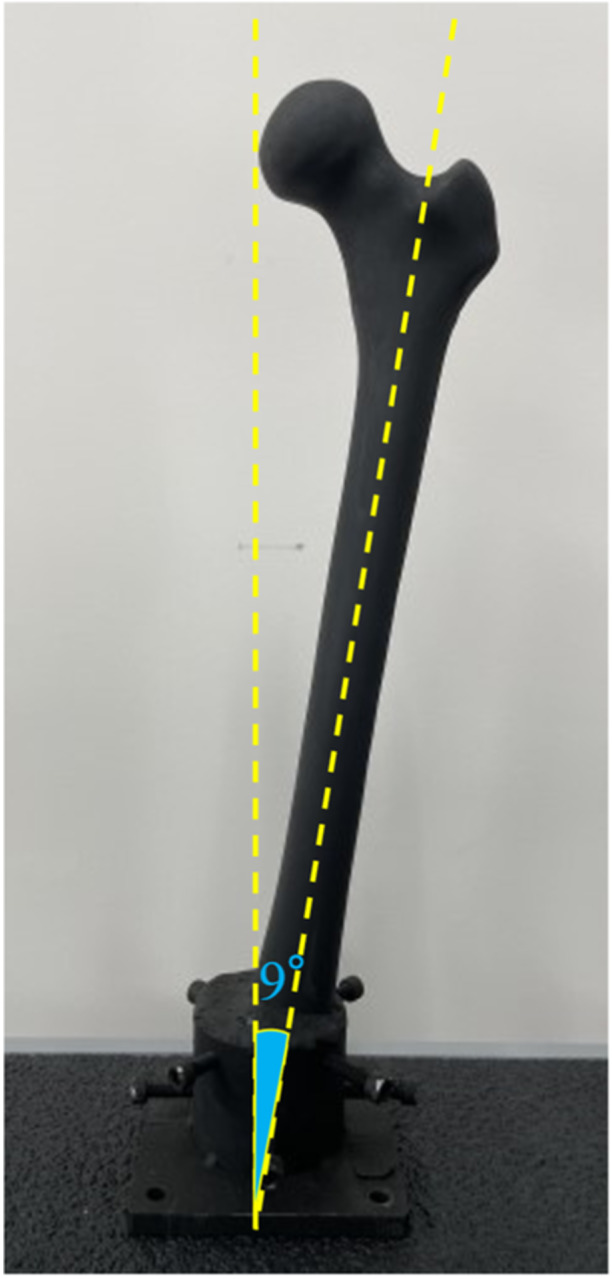
The setting of a simulated bone.

Two simulated femurs were used (nos. 1 and 2). The distal portion of the simulated femur was excised and fixed to a specimen holder with bolts and bone cement in the physiological adduction position (9 °adduction) in a one‐leg standing loading position. The sample surfaces were coated with a matte black heat‐resistant paint (ASAHIPEN Corporation) to maintain a constant heat emissivity (Figure 2).

**Figure 3:**
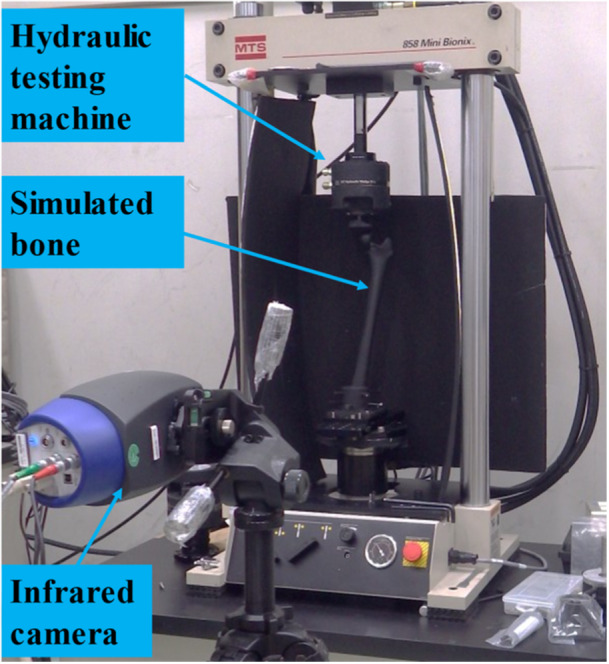
Thermoelastic stress measurement of a simulated bone.

The simulated femur was fixed to a servohydraulic testing machine (MiniBionix 858; MTS Systems Corporation), and 20–1000 N, 0.27 Hz sinusoidal compressive load was applied to the femoral head (Figure 3). Then, images of the surface stress distribution of the femur were obtained with an infrared stress measurement system (Silver450M 7500; Cedip (FLIR)) during loading; surface stress distribution images were acquired on the anterior, posterior, medial and lateral surfaces of the femur.

After measuring the simulated femur, an anthology (#5, std offset) was inserted into the femur as in the usual surgical technique for THA, and the OXINIUM femoral head was attached to the neck of the stem. The specimens were positioned in the testing machine, and images of the surface stress distribution on the simulated femur after stem insertion were acquired under the same loading conditions.

#### Stress change rate

Lateral and medial images of the stress distribution were used. The lateral and medial parts of the simulated femur were divided into seven zones according to the Gruen zones [7]. The lateral part was set to zones 1–3, and the medial part was set to zones 4–7. The mean change in the sum of principal stresses (MPa) in each zone was calculated before (intact) and after (with stem) stem insertion.

The ‘stress change rate’ of the change in the sum of principal stresses on the simulated femoral surface after stem insertion was computed for each zone using Equation (2).

(2) $$\begin{array}{l} \mathrm{Stress}\;\mathrm{change}\;\mathrm{rate}\left(\%\right)=\{(\mathrm{the}\;\mathrm{change}\;\mathrm{in}\;\mathrm{the}\;\mathrm{sum}\;\mathrm{of}\;\mathrm{principal}\;\mathrm{stresses}\;\mathrm{of}\;“\mathrm{With}\;\mathrm{stem}”)\\ -(\mathrm{the}\;\mathrm{change}\;\mathrm{in}\;\mathrm{the}\;\mathrm{sum}\;\mathrm{of}\;\mathrm{principal}\;\mathrm{stresses}\;\mathrm{of}\;“\mathrm{Intact}”)\}/\\ (\mathrm{the}\;\mathrm{change}\;\mathrm{in}\;\mathrm{the}\;\mathrm{sum}\;\mathrm{of}\;\mathrm{principal}\;\mathrm{stresses}\;\mathrm{of}\;\mathrm{Intact})\times 100. \end{array}$$

The stress change rate for each zone in simulated bones No. 1 and No. 2 was calculated, and the mean value was obtained.

### Bone mineral density change

#### Patients and methods

This study retrospectively analysed 106 consecutive patients who underwent THA with anthologies from 2017 to 2020. Fifty‐one patients with 58 hips were included in the study after excluding patients with severe deformity of the proximal femur (two hips), intraoperative or postoperative fractures (six hips), bone metabolism disease (one hip), chronic renal failure (two hips), osteoporosis drug use (11 hips), prior steroid therapy (11 hips) and no bone mineral density measurements (15 hips). Surgery was performed on 26 hips using the posterior approach and 32 hips using the anterolateral approach. Furthermore, all patients were rehabilitated with full weight‐bearing postoperatively. Data on sex, age at surgery, body mass index (BMI) and primary diagnosis for surgery were obtained from the medical records of the patients.

The study was conducted in accordance with the guidelines of the Declaration of Helsinki and approved by the Institutional Ethics Review Board of the University of Tsukuba (H26‐104). Written informed consent was obtained from the patients for the publication of this study.

The shape of the proximal femur was classified using the Dorr classification on preoperative plain anteroposterior radiographs [4]. Additionally, the canal flare index was measured [22] (Table 1).

**Table 1:** Patient demographics.

Patient demographics	
Number of hips	58
Number of patients	51
Sex; Female:Male	48:10:00
Mean age at surgery, years (range)	60 (40–82)
Mean body mass index at surgery (range) (kg/m^2^)	24.9 (16.4–43.5)
Primary diagnosis for surgery	
Primary osteoarthritis	8
Osteonecrosis of femoral head	4
Secondary osteoarthritis due to dysplasia	46
Dorr type; A:B:C	19:39:00
Mean canal flare index (range)	3.8 (2.5–6.1)

Dual‐energy X‐ray absorptiometry (DEXA) was performed using Discovery (Hologic). Seven regions of interest (ROI) were established around the stem based on the Gruen zone [7]. The values for each ROI were expressed as BMD values per area (g/cm^2^). BMD around the stem was measured at 1 week (baseline) and 1 year postoperatively. The BMD change rate at 1 year postoperatively, based on that of 1 week postoperatively, was calculated for each ROI using the following formula (3).

(3) $$\begin{array}{l} \text{BMD}\;\text{change}\;\text{rate}\left(\%\right)=\{(\text{BMD}\;\text{at}\;1\;\text{year})\\ -(\text{BMD}\;\text{at}\;1\;\text{week})\}/(\text{BMD}\;\text{at}\;1\;\text{week})\times 100. \end{array}$$

### Statistical analyses

The correlation between the stress change rate per zone obtained using TSA and the BMD change rate per zone 1 year after THA was calculated using Pearson's correlation coefficient. The level of significance was set at *p* < 0.05.

## RESULTS

### TSA

**Figure 4:**
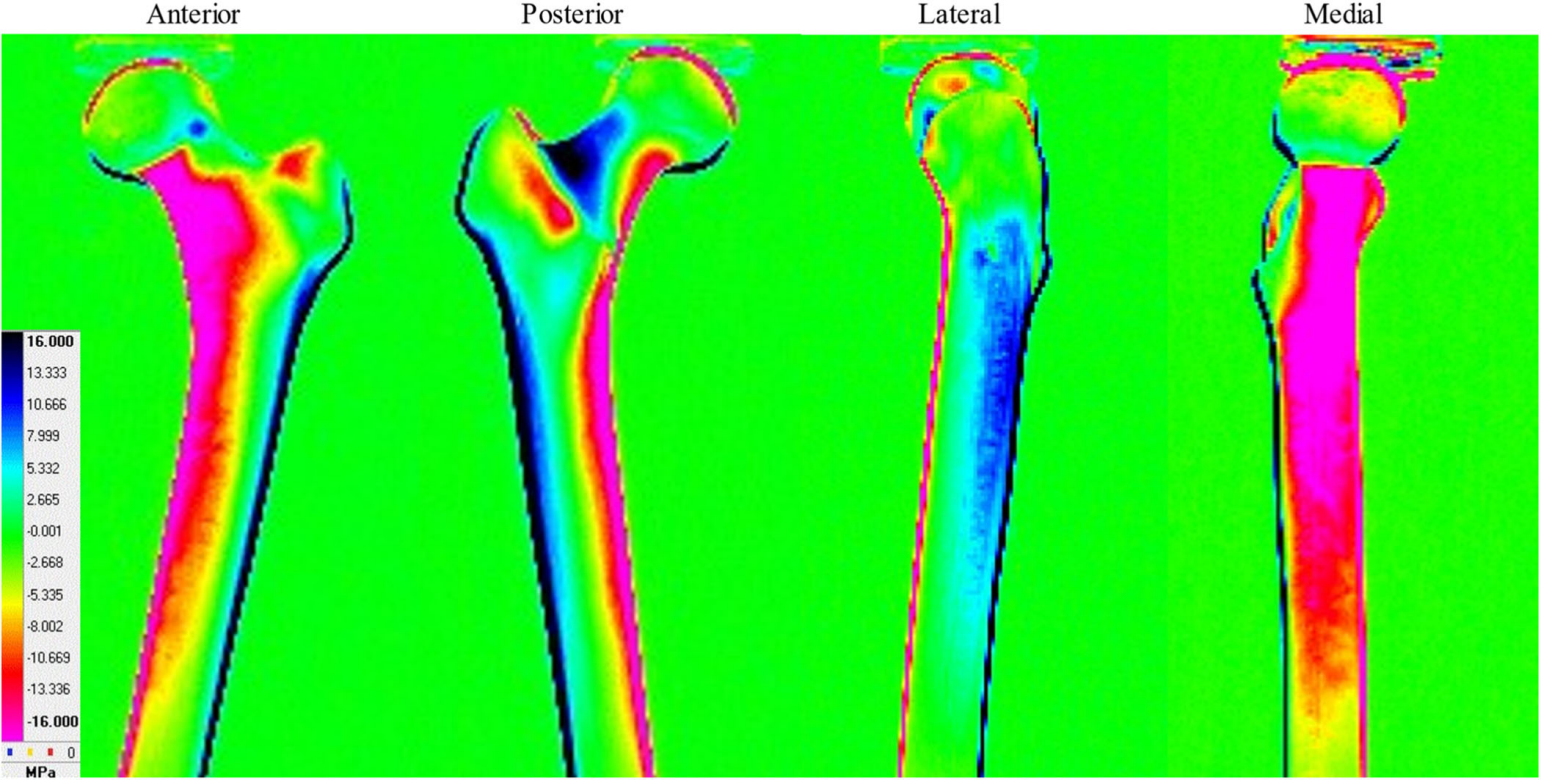
Stress distribution of simulated bone without stem (Intact No.1).

**Figure 5:**
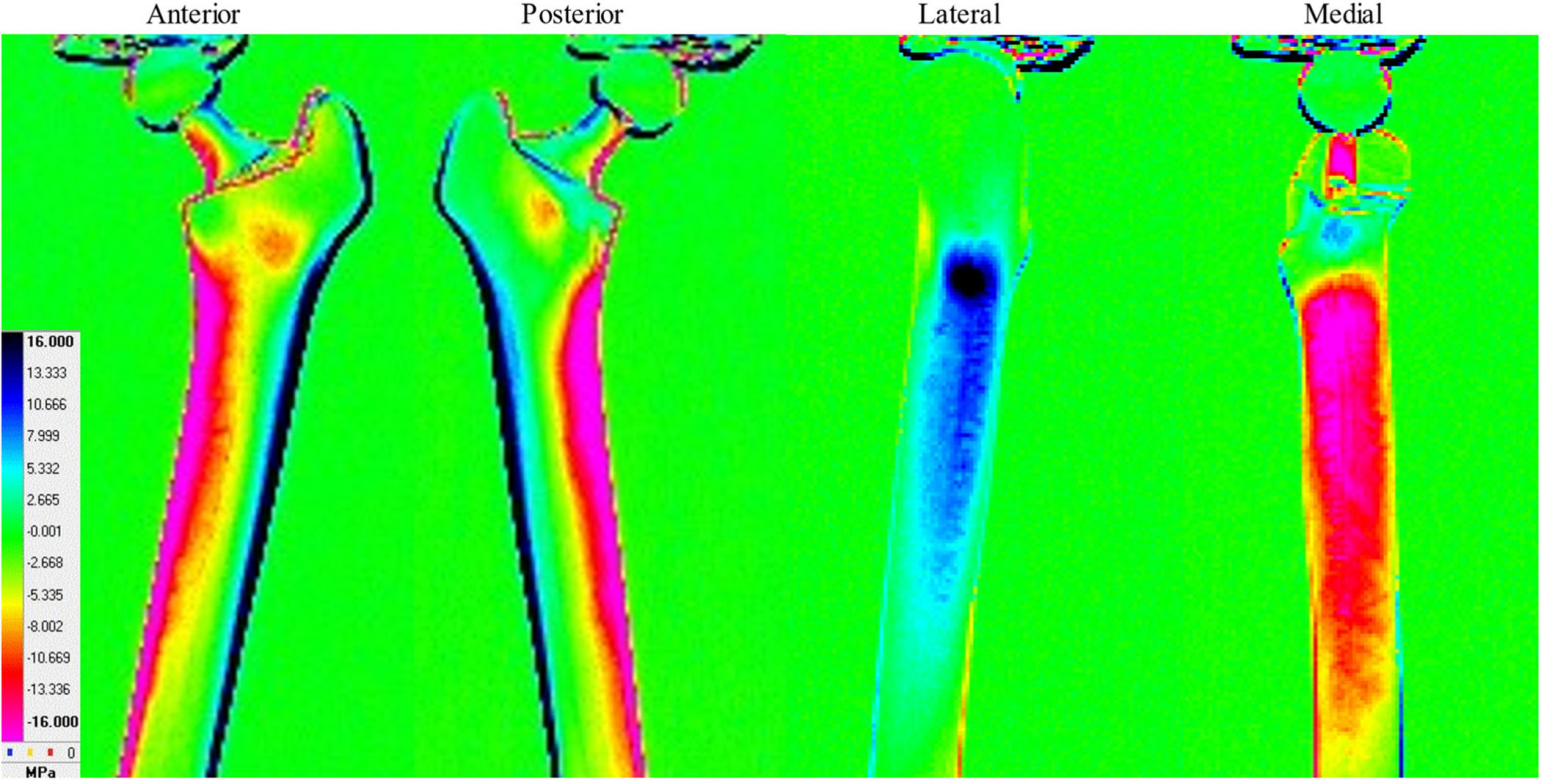
Stress distribution of simulated bone with anthology (With stem No.1).

The stress distribution in the simulated bone without a stem is depicted in Figure 4. Compressive stress (warm colour) was distributed medially from the neck to the diaphysis, and tensile stress (cold colour) was distributed laterally from the tuberosity to the diaphysis. Additionally, compressive stress was distributed on the anterior and posterior surfaces of the femoral intertrochanter. The stress distribution of the simulated bone with the Anthology is depicted in Figure 5. On the medial side, compressive stress was distributed below the lesser trochanter to the diaphysis. Furthermore, weak tensile stress was observed in the calcar. On the lateral side, the tensile stress was distributed from below the lesser trochanter to the diaphysis, and strong tensile stress was distributed in the proximal region. Weak compressive stresses were distributed on the anterior and posterior surfaces of the femoral intertrochanter.

### Stress change rate

**Figure 6:**
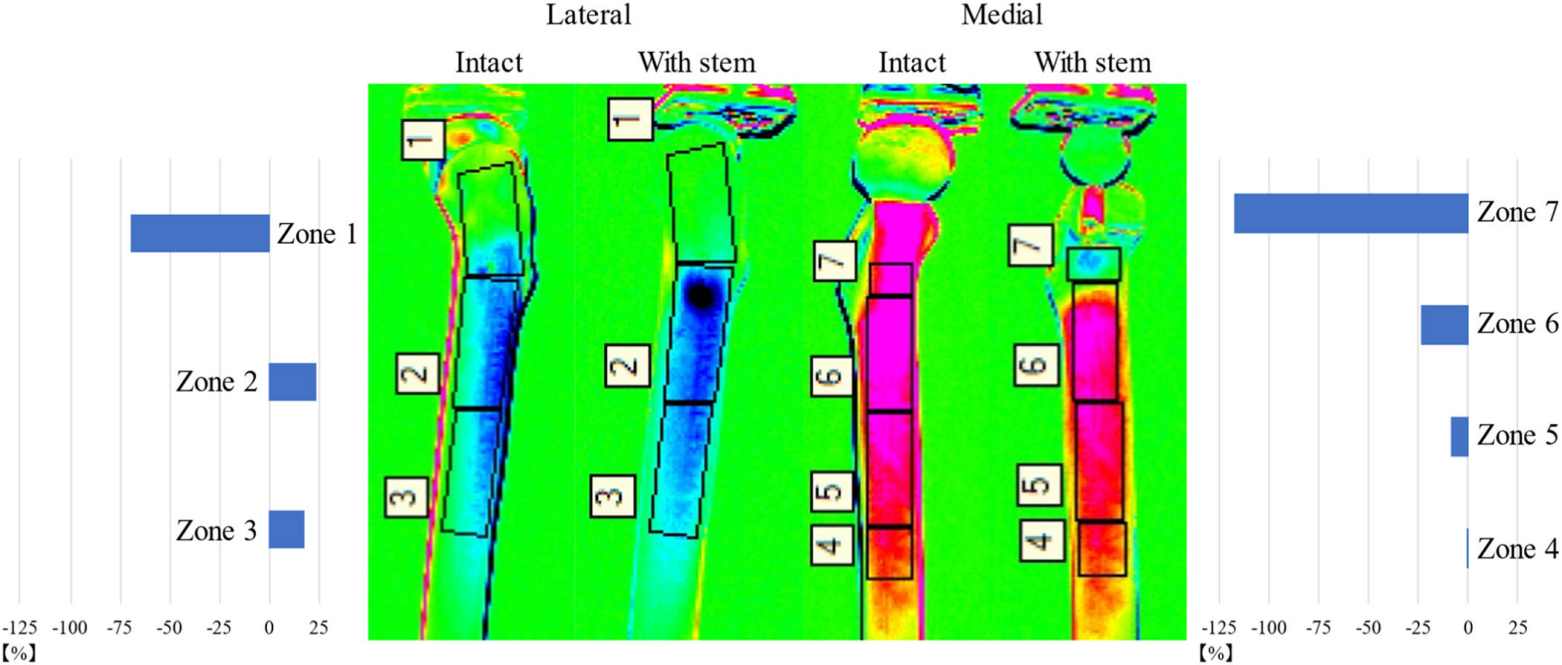
The mean stress change rate for seven zones by thermoelastic stress analysis.

The mean stress change rates for the seven zones obtained by thermoelastic stress analysis are depicted in Figure 6. In the proximal parts (zones 1 and 7), the decrease in stress was significant. The stress change rate was lower in the distal part on both the lateral and medial sides than on the other sides. In zones 2 and 3, the stress increased, whereas it decreased in zones 4 and 6.

### BMD change rate

**Figure 7:**
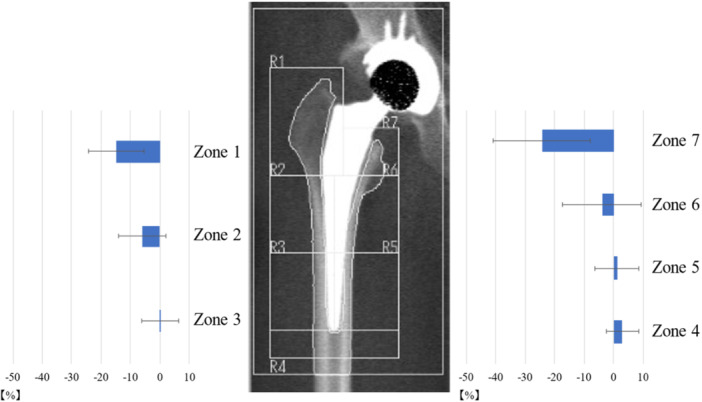
The mean bone mineral density change rate around the stem for seven zones by dual‐energy X‐ray absorptiometry.

The BMD change rate in zones 1–7 a year after THA using Anthology is revealed in Figure 7. In the proximal part (zones 1 and 7), the BMD decreased by 15%–25%; however, in zones 2–6, the BMD change rate was −6% to 3%.

### Correlation coefficient

**Figure 8:**
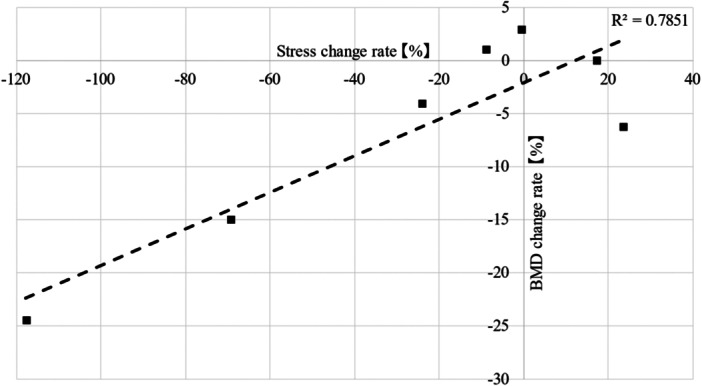
Scatter plots of stress change rate and bone mineral density (BMD) change rate. A significant correlation was observed between both stress change rate and BMD change rate (*p* = 0.008).

A significant positive correlation coefficient of 0.886 (*p* = 0.008) was observed between the rate of change in stress and BMD (Figure 8).

## DISCUSSION

In this study, we compared the results of mechanical analysis experiments by employing a tapered wedge stem with HA coating on the proximal portion with BMD changes after THA in clinical practice. In the thermoelastic stress analysis, the infrared camera captured the stress distribution changes in the entire simulated bone before and after stem insertion. The rate of stress change differed for each zone. Furthermore, there was a strong positive correlation between stress and BMD change. Mechanical analysis experiments indicated that changes in stress distribution before and after stem insertion affected the changes in BMD after THA.

In this study, a significant correlation was observed between the rate of stress change assessed using TSA before and after stem insertion and the rate of change in BMD 1 year after THA. Even though TSA assesses stress distribution shortly after stem insertion, it correlated with bone remodelling occurring over the subsequent year postimplantation. The key reason behind this lies in our focus on ‘stress change rate’ rather than ‘stress distribution’. We observed a trend where increased stress in TSA corresponded to increased BMD in the respective zones, whereas decreased stress correlated with a decline in BMD. Furthermore, this study's relevance was emphasized by its investigation of the early postoperative period of 1 year, characterized by ongoing bone remodelling with relatively minimal occurrences of bone atrophy or thickening. This temporal matching may have contributed to the high correlation observed; therefore, further studies are needed to comprehensively examine these dynamics, as bone remodelling and changes in stress distribution are expected to continue for a long period after THA.

The best feature of thermoelastic stress analysis is that only mechanical test allows noncontact evaluation of the stress distribution over the entire simulated femoral surface under load. Reports indicate the usefulness of this technique for analysing the stress distribution in a simulated femur after stem insertion. The stress distribution differs depending on the type of stem, and the contact condition of the stem influences stress distribution [13, 29]. In addition, as this method can measure the stress at any point or area on the surface, it may be compared and validated using FEA and strain gauge methods [3, 31].

In the TSA, the stress distribution changes in the proximal medial region of the simulated femur were the largest before and after stem insertion. High compressive stress was distributed from the neck to the diaphysis before the stem insertion, whereas weak tensile stress was distributed in the calcar after the stem insertion. This indicates that the ‘direction’ and ‘magnitude’ of the stress varied largely before and after the stem insertion. We considered that stress was transferred distally from the fixed areas of the stem in contact with the cortical bone and that stress shielding occurred proximal to the fixed areas [10]. Additionally, we hypothesized that this major change in stress distribution occurred after THA, which led to a decrease in the BMD in zone 7.

The Anthology is characterized by a tapered wedge‐type stem with a proximal HA coating and a proximally fixed stem [16]. Good short‐term and medium‐term results have been reported [2, 15, 20]. In previous reports, the BMD change rate around the stem at 1 year postoperatively was reported to be a 10%–18% decrease in proximal zones 1 and 7 and a 1%–6% increase in zones 2–6 [18]. This previous study used BMD at 2 months postoperatively as baseline; therefore, we considered the proximal BMD loss at 1 year postoperatively to be less than that in this study. However, this was similar to the results of the present study in that the proximal part of the stem decreased BMD, whereas the diaphysis maintained it. Similar BMD changes have been reported in other tapered wedge‐type stems [14]. The trend in BMD changes in this study was consistent with that in previous reports.

Using FEA, the correlation between stress distribution after THA and changes in BMD has been reported in several studies [6, 10, 11, 23]. These studies that use patient‐specific CT data and cadaver bones are very useful; however, their limitation is that they are only simulation studies. The present study differs from these studies in that it applies loads and evaluates the stress distribution in the entire simulated bone. The high correlation between the stress change rate and BMD change rate suggests that this method may be useful for predicting stress distribution and BMD changes after THA. Furthermore, it may be useful in predicting the progress of implants already in use, as well as in confirming the stress distribution by testing implants in the development stage before use.

This study has several limitations. First, only one type of simulated femur was used in TSA. In this study, only femurs that mimicked adult males were used. Currently, a simulated femur that mimics osteoporosis is commercially available, and experiments must be conducted to verify the difference in stress distribution in the future. Second, the loading condition was only vertical loading of the bone head. In FEA, tensile stresses such as those on the abductor muscles may be added to the greater trochanter. In particular, in Gruen zone 1, the tensile stress has a significant influence on the stress distribution. Another limitation was that only the surface stress of the simulated bone could be evaluated using TSA. We expect that these may be complemented by future analyses combined with FEA.

## CONCLUSION

We compared femoral stress using TSA with BMD changes after THA. In the TSA, stress changes before and after stem insertion were highly correlated with changes in BMD 1 year after THA. Using this method, it is possible to simulate stress distribution after THA and predict postoperative BMD changes. Furthermore, it may be useful in predicting the progress of implants already in use, as well as in confirming the stress distribution by testing implants in the development stage before use. Combined with FEA, a more detailed stress distribution analysis will also be possible.

## AUTHOR CONTRIBUTIONS

**Ryunosuke Watanabe**: Conceptualization; investigation; writing—original draft. **Hajime Mishima**: Conceptualization; supervision; writing—review and editing. **Hironori Takehashi**: Investigation. **Hiroshi Wada**: Conceptualization. **Sho Totsuka**: Analysis of the data; wrote the manuscript. **Tomofumi Nishino**: Writing—review and editing. **Masashi Yamazaki**: Supervision.

## CONFLICT OF INTEREST STATEMENT

The authors declare no conflict of interest.

## ETHICS STATEMENT

The study was conducted in accordance with the guidelines of the Declaration of Helsinki and approved by the Institutional Ethics Review Board of our institute (H26‐104). Written informed consent was obtained from the patients for the publication of this study. Written informed consent was obtained from the patients for the publication of this study.

## Data Availability

The data sets used and/or analysed in the current study are available from the corresponding author upon reasonable request.
